# Perioperative Probiotic Supplementation to Reduce Postoperative Infection and Inflammation in Children and Neonates Undergoing Gastrointestinal Surgery: A Systematic Review and Meta-Analysis

**DOI:** 10.3390/life16040569

**Published:** 2026-03-31

**Authors:** Amani N. Alansari, Marwa Messaoud, Suzie Khogeer, Myriam Ben Fredj, Mohamed Sayed Zaazouee, Amine Ksia

**Affiliations:** 1 Department of Pediatric Surgery, Hamad Medical Corporation, Doha 3050, Qatar; 2 Pediatric Surgery Department, Fattouma Bourguiba University Hospital, Monastir 5000, Tunisia; marwa.mesaoud@gmail.com (M.M.); myriam.benfredj@yahoo.fr (M.B.F.); amineks@yahoo.fr (A.K.); 3 Faculty of Medicine of Monastir, University of Monastir, Monastir 5000, Tunisia; 4 Imam Abdulrahman Bin Faisal, Ministry of National Guard Health Affairs, Dammam 22490, Saudi Arabia; drkhogeer@gmail.com; 5 Faculty of Medicine, Al-Azhar University, Assiut 21526, Egypt; mohamedzaazouee.stu.6.44@azhar.edu.eg

**Keywords:** pediatric surgery, neonatal surgery, postoperative infections, gut microbiota, inflammation

## Abstract

Background: Gastrointestinal surgery in children and neonates carries high risks of postoperative infections, inflammation, and delayed recovery. Probiotics may help restore gut microbial balance and improve clinical outcomes. This study systematically evaluates the effects of probiotic supplementation on postoperative outcomes in pediatric and neonatal gastrointestinal surgery. Methods: A systematic review and meta-analysis was conducted following Cochrane and PRISMA guidelines. PubMed, Scopus, Cochrane, and Web of Science were searched for randomized controlled trials (RCTs) and observational studies comparing probiotics with placebo, standard care, or surgery alone in children and neonates. Meta-analyses were performed using R programming, with pooled effect estimates reported alongside 95% confidence intervals (CI) and corresponding *p*-values. Results: Eight studies (6 RCTs and two cohorts) with 437 patients were included. Probiotics significantly reduced postoperative infections (risk ratio = 0.49, 95% CI: 0.26–0.92, *p* = 0.027) and C-reactive protein elevation (risk ratio = 0.42, 95% CI: 0.26–0.68, *p* < 0.001) and increased *Bifidobacterium* abundance (standardized mean difference = 0.84, 95% CI: 0.51–1.17, *p* < 0.001). No significant effect was seen on hospital length of stay or *Lactobacillus* concentrations. Qualitative synthesis indicated improvements in bowel function, immune markers, and growth in selected neonatal populations. Conclusions: Probiotics, particularly *Bifidobacterium species*, may reduce postoperative infections and inflammation in pediatric gastrointestinal surgery; however, the evidence is based on a limited number of studies and small pooled analyses, and larger well-designed trials are needed to confirm these findings.

## 1. Introduction

Gastrointestinal surgery in children and neonates is particularly challenging, not only because operating on small and developing bodies is technically demanding, but also because it places significant stress on their bodies [1,2]. Postoperative complications, including infections [3], inflammation [4], and delayed recovery [5], are major concerns that can prolong hospital stay, increase healthcare costs, and, most importantly, affect the well-being of vulnerable pediatric patients [6]. While advances in surgical techniques, anesthesia, and perioperative care have improved outcomes, managing the delicate balance of the gut environment remains an underexplored yet critical aspect of recovery [7].

The human gut hosts a complex microbial ecosystem that functions almost like an organ in itself, influencing digestion, immune function, and even systemic inflammatory responses [8]. In children and neonates, this microbial community is still developing and thus particularly sensitive to disturbances [9]. Surgical stress, anesthesia, perioperative antibiotics, and temporary fasting can all disrupt the gut microbiota, creating a state of imbalance known as dysbiosis [10]. Dysbiosis can increase susceptibility to infections, impair nutrient absorption, and exacerbate systemic inflammatory responses, making recovery more complicated [11]. Emerging evidence suggests that interventions aimed at supporting the gut microbiota could decrease these risks and improve clinical outcomes [12].

Postoperative complications remain a significant concern in pediatric gastrointestinal surgery. Global cohort studies report surgical site infection (SSI) rates ranging from 6.3% in high-income settings to 24.7% in low-income settings [13], highlighting a substantial burden of postoperative morbidity in children undergoing gastrointestinal procedures. Large population-based analyses have shown that postoperative complications occur in thousands of pediatric gastrointestinal surgical hospitalizations and are associated with prolonged hospital stays and increased healthcare costs [14].

Probiotics, live microorganisms that provide health benefits when given in adequate amounts, have attracted interest for restoring gut balance and supporting postoperative recovery [15]. They can inhibit harmful bacteria, produce antimicrobial compounds, strengthen the intestinal barrier, and modulate the immune system [16]. While these effects are well-studied in adults, children and neonates differ in gut microbiota, immune development, metabolism, and surgical responses, raising questions about whether adult findings apply to younger populations [9].

However, current pediatric probiotic studies remain limited by small sample sizes, heterogeneous surgical populations, inconsistent findings across outcomes, and a lack of standardized probiotic strains, dosing regimens, and timing of administration, which complicates interpretation and clinical translation. Some studies in pediatric surgical settings suggest probiotics can reduce infections, lower inflammation, and improve bowel function [17,18,19]. Given the growing recognition of the gut microbiota’s role in recovery, a systematic review of probiotics in this population is timely. This study evaluates the effects of probiotic supplementation on postoperative outcomes in children and neonates undergoing gastrointestinal surgery, aiming to clarify benefits, limitations, and gaps to guide clinical practice and future research.

## 2. Materials and Methods

This meta-analysis was performed following the methodological standards outlined in the Cochrane Handbook for Systematic Reviews of Interventions and is reported in line with the PRISMA (Preferred Reporting Items for Systematic Reviews and Meta-Analyses) guidelines [20,21]. The protocol was registered in PROSPERO (CRD420251208620).

### 2.1. Search Strategy

A systematic literature search was conducted across PubMed, Scopus, Cochrane, and Web of Science to identify studies evaluating the effects of probiotics in pediatric and neonatal patients undergoing gastrointestinal surgery. Search terms included combinations of “probiotics,” “*Lactobacillus*,” “*Bifidobacterium*,” “neonate,” “child,” “gastrointestinal surgery,” and related keywords. The search was limited to English-language articles published from inception to October 2025. Reference lists of relevant studies and reviews were also screened to identify additional eligible studies. The detailed search strategy is shown in Table S1.

### 2.2. Eligibility Criteria

Studies were considered eligible if they included neonates or children undergoing gastrointestinal surgery and investigated any probiotic supplementation, such as *Lactobacillus* or *Bifidobacterium species*, compared with placebo, standard care, or surgery alone. Outcomes of interest included postoperative infections, hospital length of stay, C-reactive protein (CRP) elevation, changes in gut microbiota, bowel function, and other clinically relevant postoperative measures. Both randomized controlled trials (RCTs) and observational studies were included. Studies were excluded if they were review articles, editorials, animal studies, or non-English publications.

### 2.3. Study Selection

All retrieved records were imported into EndNote version 20, and duplicates were removed. Titles and abstracts were screened for relevance, followed by full-text assessment for eligibility. Discrepancies were resolved through discussion between two independent reviewers.

### 2.4. Data Extraction

Data were extracted by two reviewers using a standardized form, including study characteristics (country, study design), participant demographics (age, sex, weight), surgical indication, probiotic type and regimen, timing and duration of administration, and outcomes of interest. Any disagreements were resolved by consensus.

### 2.5. Risk of Bias Assessment

The quality of included RCTs was assessed using the revised Cochrane Risk of Bias tool (ROB 2), which evaluates five domains: randomization, deviations from intended interventions, outcome measurement, missing data, and selective reporting [22]. Each domain was rated as low, high, or some concerns, and an overall risk of bias judgment was assigned to each study.

Retrospective cohort studies were appraised using the Newcastle–Ottawa Scale (NOS), which assesses study quality based on three key components: selection of participants, comparability of cohorts, and ascertainment of outcomes. Stars are awarded for each item within these domains to reflect methodological strength, with higher scores indicating lower risk of bias [23].

The quality of evidence for main outcomes was assessed using the Grading of Recommendations Assessment, Development and Evaluation (GRADE) approach.

### 2.6. Quantitative Synthesis

For outcomes reported in two or more studies, meta-analyses were performed using fixed-effects models for homogeneous data and random-effects models for heterogeneous data. Dichotomous outcomes were expressed as risk ratios (RR), while continuous outcomes were analyzed using mean differences or standardized mean differences (SMD). Heterogeneity was evaluated using both Cochran’s Q test and the I^2^ statistic, with a Q test *p*-value > 0.1 indicating homogeneity and the I^2^ value quantifying the extent of heterogeneity. When heterogeneity was present, random-effects models and sensitivity analyses were applied to explore potential sources; if heterogeneity remained unexplained, it was reported as unresolved. Pooled effect estimates are presented with 95% confidence intervals (CI) and corresponding *p*-values. All statistical analyses were performed using R programming.

Subgroup analyses were conducted based on timing of probiotic initiation, defined as early (within 1–3 days post-surgery) and delayed (approximately 5–10 days post-surgery), as well as by patient age group (older children vs. neonates), to evaluate differences in postoperative infection outcomes. Publication bias for postoperative infection outcomes was assessed visually using a funnel plot.

### 2.7. Qualitative Synthesis

For outcomes that could not be included in a meta-analysis, a descriptive synthesis was conducted to summarize key findings, including effects on gut microbiota composition, immune markers, defecation function, postoperative complications, and growth outcomes.

## 3. Results

**Figure 1:**
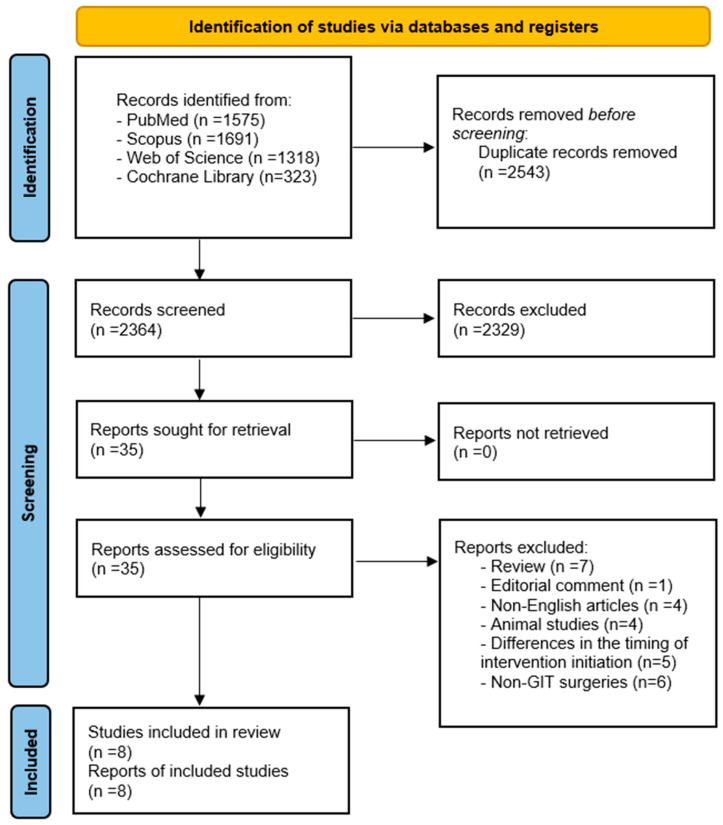
PRISMA flow diagram of study selection.

Our search retrieved 4907 records from PubMed (1575), Scopus (1691), Cochrane (323), and Web of Science (1318). After removing 2543 duplicates, 2364 records were screened by title and abstract, excluding 2329 as irrelevant. Thirty-five full-text reports were assessed for eligibility, with exclusions made for review articles (7), editorial comments (1), non-English articles (4), animal studies (4), differing intervention timing (5), and non-GIT surgeries (6). Finally, eight studies met all criteria and were included in the systematic review synthesis [17,18,19,24,25,26,27,28] (Figure 1).

### 3.1. Baseline and Summary of the Included Studies

Across the eight included studies comprising 437 children and neonates from China, the USA, Japan, Poland, Australia, and Bangladesh, six were RCTs [18,19,25,26,27,28] and two were retrospective cohort studies [17,24]. Probiotic interventions most commonly included *Lactobacillus rhamnosus* and various *Bifidobacterium species*, administered either preoperatively or with the initiation of first oral feeds, for durations ranging from 5 to 180 days, depending on the surgical context and study objectives. Surgical indications encompassed appendectomy for complicated appendicitis, postoperative management of Hirschsprung disease following the Soave procedure, biliary atresia after Kasai portoenterostomy, gastroschisis repair, intestinal atresias, and mixed congenital anomalies among NICU populations (Table 1).

Baseline characteristics were generally comparable between probiotic and control groups across studies, with similar distributions in age, sex, and weight. The average age among pediatric participants ranged from approximately 30 to 126 months, while neonates were enrolled between 0.06 and 3.09 months of age. Further details are shown in Table 2.

### 3.2. Quality Assessment

**Figure 2:**
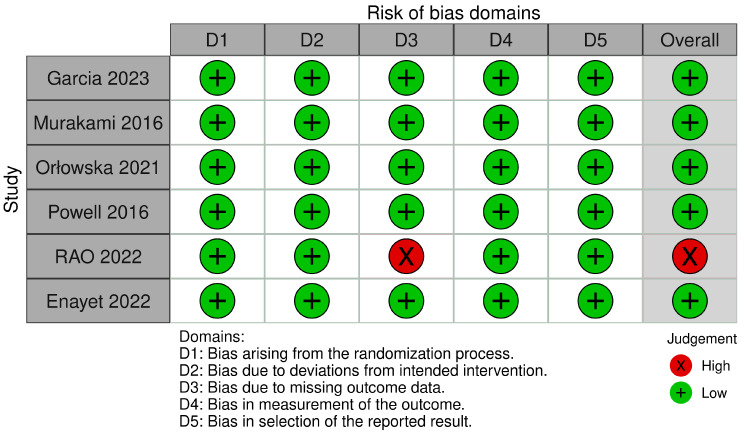
Risk of bias assessment of included RCTs (ROB 2).

The risk of bias assessment indicated that all included RCTs, except Rao et al. (2022), exhibited a low risk across all evaluated domains. Rao et al. (2022) showed a high risk of bias, specifically in the domain of missing outcome data [28] (Figure 2).

Regarding the included cohort studies, both demonstrated good overall quality, showing adequate selection, comparability, and outcome assessment [17,24] (Table S2).

The certainty of evidence for this meta-analysis ranged from high to very low according to the GRADE framework. Clinical outcomes derived from randomized controlled trials, including postoperative infection rates and hospital length of stay, were classified as high certainty with no serious risks of bias, inconsistency, or imprecision. In contrast, laboratory-based outcomes were rated as very low certainty. This is explained by the inclusion of non-randomized studies and by serious inconsistency, characterized by substantial statistical heterogeneity and discrepancies in microbiological measurement units across included studies (Table S3).

### 3.3. Quantitative Synthesis

#### 3.3.1. Postoperative Infections

**Figure 3:**
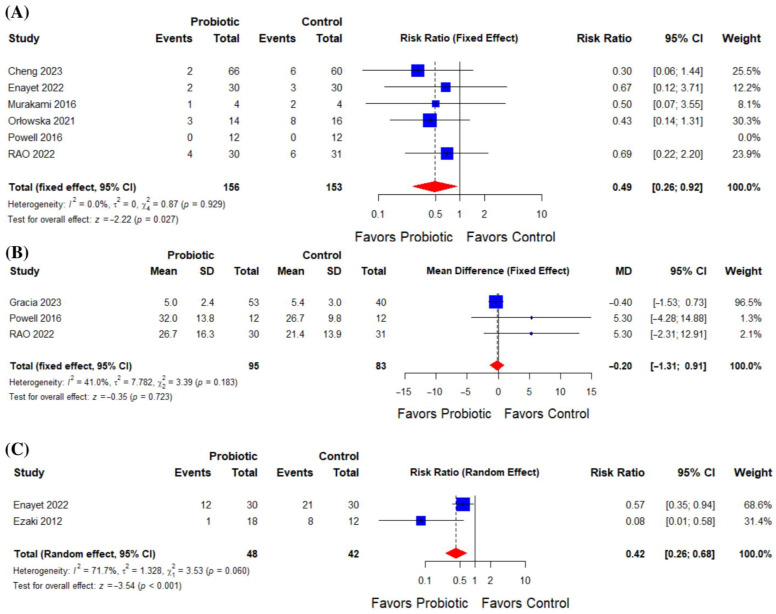
(**A**) Effect of probiotics on postoperative infections, (**B**) effect of probiotics on hospital length of stay, and (**C**) effect of probiotics on C-reactive protein (CRP) elevation.

The pooled analysis of five studies (n = 156 probiotic; n = 153 control) yielded a risk ratio of 0.49 (95% CI: 0.26 to 0.92; *p* = 0.027), indicating a significant reduction in postoperative infections among patients receiving probiotics, with no heterogeneity (I^2^ = 0%; *p* = 0.93). Notably, Powell et al. (2016) reported zero events in both groups (Figure 3A).

The funnel plot for postoperative infections appeared broadly symmetrical, suggesting no clear evidence of publication bias (Figure S1).

Subgroup analyses for postoperative infection outcomes showed that early initiation of probiotics resulted in an RR of 0.30 (95% CI: 0.06–1.44), while delayed initiation showed an RR of 0.54 (95% CI: 0.26–1.13), with no significant subgroup difference (*p* = 0.515). Age-based subgroup analysis demonstrated an RR of 0.42 (95% CI: 0.19–0.95) in older children and 0.64 (95% CI: 0.24–1.74) in neonates, with no significant subgroup difference (*p* = 0.528). Overall heterogeneity was low (I^2^ = 0%). Sensitivity analysis by study design, excluding the cohort study (Cheng 2023), showed no significant difference (RR = 0.56; 95% CI: 0.28–1.10; *p* = 0.094) (Figures S2–S4).

#### 3.3.2. Hospital Length of Stay

The pooled analysis of three studies (n = 95 probiotic; n = 83 control) showed no significant reduction in hospital stay with probiotics (mean difference −0.20 days; 95% CI: −1.31 to 0.91; *p* = 0.723), with moderate heterogeneity across studies (I^2^ = 41%; *p* = 0.18) (Figure 3B).

#### 3.3.3. C-Reactive Protein (CRP) Elevation

Analysis of two studies (n = 48 probiotic; n = 42 control) demonstrated a reduction in postoperative CRP elevation with probiotics (RR = 0.42; 95% CI: 0.26 to 0.68; *p* < 0.001), but unresolvable heterogeneity was observed (I^2^ = 71.7%; *p* = 0.06) (Figure 3C).

#### 3.3.4. *Lactobacillus* and *Bifidobacterium* Concentration

**Figure 4:**
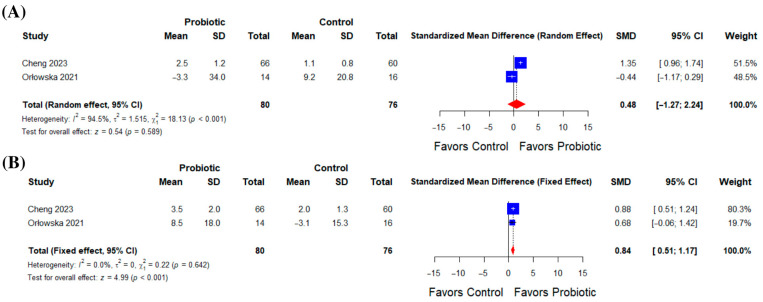
(**A**) Effect of probiotics on *Lactobacillus* concentrations, and (**B**) effect of probiotics on *Bifidobacterium* concentrations.

Analysis of two studies examining changes in *Lactobacillus* concentrations showed an insignificant effect compared to control (SMD = 0.48; 95% CI: –1.27 to 2.24; *p* = 0.589), with unresolvable heterogeneity (I^2^ = 94.5%; *p* < 0.001) (Figure 4A).

In contrast, pooling two studies for *Bifidobacterium* concentrations demonstrated a substantial increase with probiotics (SMD = 0.84; 95% CI: 0.51 to 1.17; *p* < 0.001), with minimal heterogeneity (I^2^ = 0%; *p* = 0.64) (Figure 4B).

### 3.4. Qualitative Synthesis

#### 3.4.1. Probiotics and Postoperative Outcomes

Probiotics showed several benefits in neonates and children undergoing gastrointestinal surgery. In neonates receiving *Bifidobacterium* breve, cow’s milk protein intolerance was significantly lower (6% vs. 67%), intestinal flora was improved, and immune maturation was supported [17]. In children under five, probiotics did not reduce surgical site infections or postoperative fever but indicated reduced systemic inflammation, while total white blood cell and neutrophil counts remained unchanged [25].

In children with Hirschsprung disease undergoing the Soave procedure, combined *Bifidobacterium* and *Lactobacillus* supplementation significantly improved defecation function, with an excellent Krickenbeck score rate of 96.97% versus 85% in controls [24].

#### 3.4.2. Gut Dysbiosis and Growth

Neonates with congenital gastrointestinal surgical conditions receiving a three-strain bifidobacterial probiotic had reduced pathogenic bacterial abundance, increased Bifidobacteriaceae, higher stool short-chain fatty acid levels, and less postnatal head growth restriction, suggesting potential neurodevelopmental benefits. No probiotic-related infections occurred [28].

#### 3.4.3. Microbiota Changes in Urgent Surgery

In neonates receiving *Bifidobacterium* animalis LKM512, surgery altered intestinal microbiota irrespective of probiotics. Probiotic use did not clearly enhance beneficial bacteria or clinical outcomes such as weight gain or infection prevention, emphasizing the need for further research on optimal timing and dosing [26].

#### 3.4.4. Antibiotic-Associated Diarrhea

**Figure 5:**
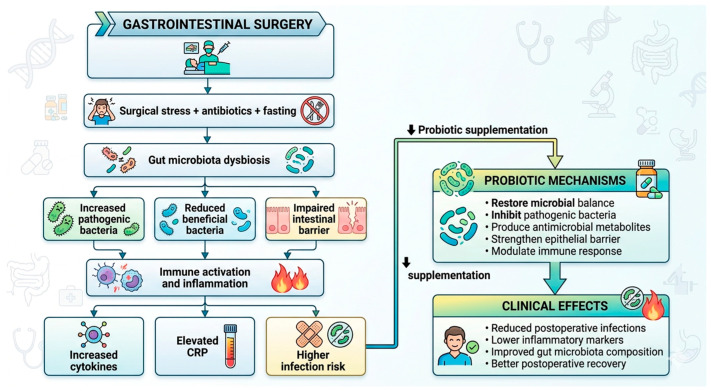
Probiotics as a strategy to mitigate postoperative inflammation, barrier dysfunction, and complications.

Among pediatric patients undergoing appendectomy for complicated appendicitis, *Lactobacillus rhamnosus GG* did not notably affect diarrhea incidence, though diarrhea peaked earlier in the probiotic group [18]. No adverse events were reported. Figure 5 shows a flowchart for the mechanistic approach.

## 4. Discussion

Our meta-analysis reveals that probiotic supplementation significantly reduces postoperative infections in children and neonates undergoing gastrointestinal surgery, cutting the risk by approximately 51%. This protective effect appeared consistently across the studies we examined, giving us confidence in the finding. We also found that probiotics significantly reduced C-reactive protein elevation, a marker that tells us how much inflammation is occurring in the body. Interestingly, *Bifidobacterium* bacteria increased significantly in children receiving probiotics, while *Lactobacillus* changes were less consistent. However, probiotics did not shorten hospital stays, suggesting that while they help prevent certain complications, they do not accelerate overall recovery time. The reduction in C-reactive protein and changes in microbiota, including increased *Bifidobacterium*, were based on only two studies and should therefore be interpreted cautiously as exploratory findings. Subgroup analyses suggested a consistent reduction in postoperative infections with probiotics across both initiation timing and age groups.

These findings align with the known physiological effects of surgery on the gastrointestinal tract. Surgical interventions disrupt intestinal microbial homeostasis through multiple mechanisms, including mucosal injury, perioperative antibiotic exposure, altered intestinal perfusion, and temporary cessation of enteral feeding [29]. Such disturbances favor the overgrowth of pathogenic microorganisms, increasing the risk of infection [30]. Probiotic supplementation may decrease these effects by competitively inhibiting pathogen colonization, producing antimicrobial metabolites, enhancing epithelial barrier integrity, and modulating immune responses to attenuate postoperative inflammation [31,32]. The observed reduction in C-reactive protein likely reflects this immunomodulatory action, suggesting that probiotics influence host inflammatory pathways rather than alleviating clinical symptoms [16].

The beneficial role of *Bifidobacterium* species warrants particular attention. These commensal bacteria predominate in the intestinal microbiota of infants and exhibit strong adhesion to the intestinal mucosa of young children [33]. Through the production of short-chain fatty acids, *Bifidobacterium* supports epithelial cell nutrition, reinforces mucosal barrier function, and creates an environment unfavorable to pathogenic bacterial proliferation [34].

Our findings are generally consistent with previous research on probiotics in surgical patients, though key differences emerge. Kasatpibal et al. (2017) conducted a network meta-analysis of 2952 adults undergoing elective surgery and found that synbiotics most effectively reduced surgical site infections, pneumonia, sepsis, hospital stay, and antibiotic use [35]. While their results showed overall reductions in postoperative complications, our pediatric-focused analysis demonstrated a greater 51% reduction in infections. This may reflect that children’s developing gut microbiota respond more readily to probiotic modulation than the more stable microbial communities in adults [36].

Similarly, Yang et al. (2017) reported that probiotics and synbiotics reduced infectious complications and shortened hospital stay by 3.2 days in adults after gastrointestinal surgery [37]. More recent meta-analyses in colorectal cancer surgery have similarly reported that probiotics reduce infections but show minimal or no effect on hospital length of stay [38]. Our analysis found no significant reduction in hospital stay, suggesting that pediatric discharge decisions depend on broader clinical and social factors beyond infection control [39].

Goldenberg et al. (2017) reported a reduction in Clostridioides difficile–associated diarrhea among patients receiving probiotics with antibiotics, but their cohort excluded surgical patients [40]. This larger effect may indicate that surgical stress, anesthesia, altered gut perfusion, and feeding interruptions limit probiotic efficacy postoperatively [41].

Recent evidence further supports the potential role of probiotics in improving postoperative outcomes. Updated systematic reviews and umbrella meta-analyses have reported that perioperative probiotics or synbiotics can reduce postoperative infections and other complications across various gastrointestinal surgeries, likely through modulation of gut microbiota and immune responses [42]. Similarly, Wu et al. (2025) reported that perioperative probiotics were associated with reduced postoperative infections and improved gut microbiota balance; however, most included studies involved adult surgical populations, highlighting the limited pediatric evidence [43].

Probiotics may exert beneficial effects through several molecular pathways. *Lactobacillus* and *Bifidobacterium* species can inhibit pathogenic bacteria via production of bacteriocins, lactic acid, and other antimicrobial metabolites [31,32]. They also enhance intestinal barrier function by regulating tight junction proteins and reducing epithelial permeability [32]. In addition, probiotics modulate host immune responses through pathways such as NF-κB signaling, promoting anti-inflammatory cytokine production while suppressing pro-inflammatory mediators [16,31]. These mechanisms may contribute to reduced postoperative inflammation and infection risk following gastrointestinal surgery. These mechanisms may be particularly relevant in neonates, whose immune systems and gut microbiota are still developing. NICU-related factors such as antibiotics and parenteral nutrition can further disrupt microbial balance. Although no probiotic-related infections were reported in the included studies, safety considerations remain important in vulnerable neonatal populations.

Our findings have important practical implications. The significant reduction in postoperative infections suggests that probiotics could become part of routine care for children undergoing gastrointestinal surgery, particularly in hospitals or regions where infection rates are high [44]. Probiotics are inexpensive and widely available, and our review found them to be safe, with no probiotic-related infections reported in any of the included studies [45]. These characteristics make them attractive for widespread use. However, the lack of impact on hospital length of stay reminds us that probiotics address only one aspect of recovery. Doctors should view them as an addition to—not a replacement for—careful surgical technique, appropriate antibiotic use, and comprehensive postoperative care [46]. The reduction in inflammatory markers we observed hints at benefits that might extend beyond infection prevention, potentially including reduced scar tissue formation, better wound healing, and improved long-term gut function [47], though these outcomes need dedicated research.

### 4.1. Strengths and Limitations of Our Study

Our study has several considerable strengths. It is the first systematic review to focus specifically on the effects of probiotics in pediatric and neonatal surgical populations, addressing an important gap in the literature. We followed well-established international standards for systematic reviews, conducted comprehensive searches across multiple major databases, and used a transparent, reproducible process with two independent reviewers at every stage. The methodological quality of included studies was formally assessed using established appraisal tools. Moreover, the consistent finding of reduced postoperative infections across different studies increases confidence in the reliability of this result.

However, several limitations should be acknowledged. First, only eight studies met our inclusion criteria, and several pooled analyses were based on only two or three studies, which limits the robustness of the estimates and makes the results more sensitive to the influence of individual studies. Second, substantial heterogeneity existed across studies in probiotic strains, dosages, timing, duration of administration, patient age groups, and surgical procedures—including appendectomy, Hirschsprung disease surgery, biliary atresia repair, and surgeries for congenital gastrointestinal anomalies—which differ in complexity and baseline infection risk, limiting the ability to draw precise clinical recommendations or attribute outcomes solely to probiotics. Another limitation is the difficulty in establishing causality due to variability in study designs and the lack of long-term longitudinal and large interventional studies. Finally, most studies originated from a limited number of countries, and only English-language publications were included, introducing a potential language bias.

### 4.2. Conclusions

This meta-analysis suggests that probiotics may help reduce postoperative infections and inflammation in children and neonates undergoing gastrointestinal surgery, indicating potential value as part of perioperative care. The evidence appears more consistent for *Bifidobacterium* species.

However, these findings should be interpreted cautiously because the evidence is derived from a small number of studies with limited pooled data. Further large, well-designed multicenter trials are needed to confirm these effects and clarify the optimal strains, timing, dosage, and duration of probiotic use.

### 4.3. Recommendations

Future research should focus on large, multi-center studies that compare specific probiotic formulations in clearly defined groups of surgical patients, measure outcomes using standardized methods, and follow patients long-term to assess growth, development, and quality of life. Advanced genetic techniques for studying gut bacteria could help identify which patients are most likely to benefit from probiotics. Until better evidence becomes available, probiotics should be considered as a low-risk addition to standard care in pediatric gastrointestinal surgery, especially when infection risk is high, while maintaining realistic expectations about overall impact on recovery.

## Figures and Tables

**Table 1:** Summary of the included studies.

ID	Study Design	Country	Sample Size	Population Details	Probiotic			Control Arm Details	Duration of Intervention (Days)	Specific Surgical Condition	Type of Surgery	Primary Outcomes Measured
					Strain (s)	Dose	Timing of Initiation					
Cheng 2023	Retrospective cohort	China	126	Children	1-*Bifidobacterium* spp., 2-*Lactobacillus* spp., 3-*Streptococcus thermophilus*	9 tablets/day	Postoperative day 3	Antibiotics	90	Hirschsprung disease	Soave radical operation	Clinical Efficacy and Total Response Rate
Garcia 2023	Randomized controlled trial	USA	93	Children	*Lactobacillus rhamnosus* GG	10 billion CFU twice daily	Post-appendectomy	Antibiotics plus placebo made of methylcellulose	5	Complicated appendicitis	Appendectomy	Length of Stay
Enayet 2022	Randomized controlled trial	Bangladesh	60	Children	1-*Lactobacillus acidophilus*, 2-*Lactobacillus bulgaricus*, 3-*Bifidobacterium bifidum*, 4-*Fructo-oligosaccharides*	<1 year: 2 capsules/day; >1 year: 3 capsules/day (*L. acidophilus* 2 billion, *L. bulgaricus* 1 billion, *B. bifidum* 1 billion, FOS 100 mg per capsule)	3 days preoperatively	Antibiotics (3rd generation cephalosporin and metronidazole)	10	1-Enterostomy closures (colostomy, ileostomy, ileocolostomy), 2-Abdominoperineal pull-through, 3-Excision of mesenteric cyst	1- Enterostomy closures, 2- Abdominoperineal pull through	Surgical Site Infection
Rao 2022	Randomized controlled trial	Australia	61	Neonates	1-*Bifidobacterium breve M-16V*, 2-*Bifidobacterium longum* subsp. *infantis M-63*, 3-*Bifidobacterium longum* subsp. *longum BB536*	3 × 10^9^ CFU/day	-	Antibiotics plus placebo made of Maltodextrin	16	1-Congenital Diaphragmatic Hernia, 2-Gastroschisis, 3-Oesophageal Atresia with Tracheo-Oesophageal Fistula, 4-Congenital Duodenal Obstruction, 5-Ileal Atresia, 6-Jejuno-Ileal Atresia, 7-Imperforate anus, 8-Exomphalos, 9-Malrotation, 10-Colon perforation, 11-Hirschsprung disease	-	Sum of Relative Abundance of Potentially Pathogenic Families
Orłowska 2021	Randomized controlled trial	Poland	30	Children	*Lactobacillus rhamnosus* GG	6 × 10^9^ CFU twice daily	Initiation of oral feeding	Antibiotics plus placebo made of glucose	180	Biliary Atresia	Kasai hepatoportoenterostomy	Diagnosis of Bacterial Cholangitis
Murakami 2016	Randomized controlled trial	Japan	13	Neonates	*Bifidobacterium animalis subsp. lactis LKM512*	1 g/day (6 × 10^9^ CFU)	Initiation of oral feeding	Antibiotics (ampicillin)	15	1-Anorectal malformation, 2-Duodenal Atresia	-	Analysis of Intestinal Microbiota
Powell 2016	Randomized controlled trial	USA	24	Neonates	*Bifidobacterium longum* subsp. *infantis* ATCC 15697	10^9^ CFU twice daily	Initiation of oral feeding	Antibiotics plus placebo made of powdered elemental formula	42	1-Gastroschisis, 2-Intestinal Atresia	Gastroschisis defect repair	Composition of the Fecal Microbiota
Ezaki 2012	Retrospective cohort	Japan	30	Neonates	*Bifidobacterium breve*	0.5 mL suspension (2.5 × 10^8^ CFU) three times daily	Initiation of oral feeding	Antibiotics (cefotaxime sodium and gentamicin sulfate)	Until full enteral feeding	1-Small intestinal atresia, 2-Localized intestinal perforation	Small intestine surgery	Cow’s Milk Protein Intolerance

**Table 2:** Baseline characteristics of the included studies.

ID	Study Groups	Age (Months), Mean (SD)	Gender (Male), N (%)	Birth Weight (kg), Mean (SD)	Baseline Weight (kg), Mean (SD)
Cheng 2023	Probiotic Group (n = 66)	30.36 (9.48)	38 (57.58%)	-	12.8 (1.6)
	Control Group (n = 60)	29.7 (11.4)	37 (61.67%)		13.2 (1.5)
Garcia 2023	Probiotic Group (n = 53)	122 (42)	33 (62.26%)	-	-
	Control Group (n = 40)	126 (47)	17 (42.5%)		
Enayet 2022	Probiotic Group (n = 30)	32.4 (14.7)	14 (46%)	-	-
	Control Group (n = 30)	40.3 (17)	23 (76%)		
Rao 2022	Probiotic Group (n = 30)	0.06	-	3 (0.8)	-
	Control Group (n = 31)			2.8 (0.5)	
Orłowska 2021	Probiotic Group (n = 14)	3.09 (1.35)	-	-	4.8 (0.4)
	Control Group (n = 16)	2.911 (0.88)			4.8 (0.4)
Murakami 2016	Probiotic Group (n = 4)	-	5 (62.5%)	-	-
	Surgical Control Group (n = 4)				
	CS Group (n = 2)		2 (100%)		
	CN Group (n = 3)		0		
Powell 2016	Probiotic Group (n = 12)	-	5 (41.6%)	2.5 (0.4)	-
	Control Group (n = 12)		6 (50%)	2.5 (0.3)	
Ezaki 2012	Probiotic Group (n = 18)	0.4 (0.5)	-	1.9 (0.9)	-
	Control Group (n = 12)	0.13 (0.1)		2 (0.9)	

Control, Suspected Surgery (CS), Control Normal (CN).

## Data Availability

No new data were created in this study.

## Supplementary Materials

The following supporting information can be downloaded at: https://www.mdpi.com/article/10.3390/life16040569/s1, Table S1: Search strategy for each database; Table S2: Quality assessment of Cohort studies with the NOS tool; Table S3: GRADE assessment for main outcomes; Figure S1: Funnel plot for postoperative infection outcome; Figure S2: Subgroup analysis for postoperative infection outcome according to the timing of probiotic initiation; Figure S3: Subgroup analysis for postoperative infection outcome according to the age group; Figure S4: Sensitivity analysis for postoperative infection outcome according to study design.
