# Supplementary material for: Perioperative Probiotic Supplementation to Reduce Postoperative Infection and Inflammation in Children and Neonates Undergoing Gastrointestinal Surgery: A Systematic Review and Meta-Analysis
Source: Life (Basel). 2026 Mar 31;16(4):569. doi: 10.3390/life16040569 (PMC13117703; doi:10.3390/life16040569)

Supplementary Table 1: Search strategy for each database.

Database	Search Query	Results
**PubMed**	(pediatric*[Title/Abstract] OR paediatric*[Title/Abstract] OR child*[Title/Abstract] OR adolescen*[Title/Abstract] OR infant*[Title/Abstract] OR neonat*[Title/Abstract] OR teen*[Title/Abstract] OR preterm[Title/Abstract]) AND (probiotic*[Title/Abstract] OR synbiotic*[Title/Abstract] OR prebiotic*[Title/Abstract] OR microbio*[Title/Abstract] OR lactobacill*[Title/Abstract] OR bifidobacter*[Title/Abstract] OR flora[Title/Abstract]) AND ((surg*[Title/Abstract] OR operat*[Title/Abstract] OR postoperat*[Title/Abstract] OR perioperat*[Title/Abstract] OR anastomo*[Title/Abstract] OR resect*[Title/Abstract] OR transplant*[Title/Abstract] OR laparoscop*[Title/Abstract] OR laparotom*[Title/Abstract]) AND (digestiv*[Title/Abstract] OR gastrointest*[Title/Abstract] OR GI[Title/Abstract] OR abdomin*[Title/Abstract] OR bowel[Title/Abstract] OR intestin*[Title/Abstract] OR esophag*[Title/Abstract] OR gastr*[Title/Abstract] OR colon*[Title/Abstract] OR hepat*[Title/Abstract] OR pancrea*[Title/Abstract]))	1575
**Web of Science (WOS)**	(TI=((pediatric* OR paediatric* OR child* OR adolescen* OR infant* OR neonat* OR teen* OR preterm) AND (probiotic* OR synbiotic* OR prebiotic* OR microbio* OR lactobacill* OR bifidobacter* OR flora) AND ((surg* OR operat* OR postoperat* OR perioperat* OR anastomo* OR resect* OR transplant* OR laparoscop* OR laparotom*) AND (digestiv* OR gastrointest* OR GI OR abdomin* OR bowel OR intestin* OR esophag* OR gastr* OR colon* OR hepat* OR pancrea*)))) OR AB=((pediatric* OR paediatric* OR child* OR adolescen* OR infant* OR neonat* OR teen* OR preterm) AND (probiotic* OR synbiotic* OR prebiotic* OR microbio* OR lactobacill* OR bifidobacter* OR flora) AND ((surg* OR operat* OR postoperat* OR perioperat* OR anastomo* OR resect* OR transplant* OR laparoscop* OR laparotom*) AND (digestiv* OR gastrointest* OR GI OR abdomin* OR bowel OR intestin* OR esophag* OR gastr* OR colon* OR hepat* OR pancrea*)))	1318

**Scopus**	(TITLE ((pediatric* OR paediatric* OR child* OR adolescen* OR infant* OR neonat* OR teen* OR preterm) AND (probiotic* OR synbiotic* OR prebiotic* OR microbio* OR lactobacill* OR bifidobacter* OR flora) AND ((surg* OR operat* OR postoperat* OR perioperat* OR anastomo* OR resect* OR transplant* OR laparoscop* OR laparotom*) AND (digestiv* OR gastrointest* OR GI OR abdomin* OR bowel OR intestin* OR esophag* OR gastr* OR colon* OR hepat* OR pancrea*)))) OR ABS ((pediatric* OR paediatric* OR child* OR adolescen* OR infant* OR neonat* OR teen* OR preterm) AND (probiotic* OR synbiotic* OR prebiotic* OR microbio* OR lactobacill* OR bifidobacter* OR flora) AND ((surg* OR operat* OR postoperat* OR perioperat* OR anastomo* OR resect* OR transplant* OR laparoscop* OR laparotom*) AND (digestiv* OR gastrointest* OR GI OR abdomin* OR bowel OR intestin* OR esophag* OR gastr* OR colon* OR hepat* OR pancrea*))))	1691
**Cochrane library**	((pediatric* OR paediatric* OR child* OR adolescen* OR infant* OR neonat* OR teen* OR preterm) AND (probiotic* OR synbiotic* OR prebiotic* OR microbio* OR lactobacill* OR bifidobacter* OR flora) AND ((surg* OR operat* OR postoperat* OR perioperat* OR anastomo* OR resect* OR transplant* OR laparoscop* OR laparotom*) AND (digestiv* OR gastrointest* OR GI OR abdomin* OR bowel OR intestin* OR esophag* OR gastr* OR colon* OR hepat* OR pancrea*))):ti OR ((pediatric* OR paediatric* OR child* OR adolescen* OR infant* OR neonat* OR teen* OR preterm) AND (probiotic* OR synbiotic* OR prebiotic* OR microbio* OR lactobacill* OR bifidobacter* OR flora) AND ((surg* OR operat* OR postoperat* OR perioperat* OR anastomo* OR resect* OR transplant* OR laparoscop* OR laparotom*) AND (digestiv* OR gastrointest* OR GI OR abdomin* OR bowel OR intestin* OR esophag* OR gastr* OR colon* OR hepat* OR pancrea*))):ab	323

Supplementary Table 2: Quality assessment of Cohort studies with the NOS tool.

ID	Selection				Comparability	Outcome			Overall judgment
	D1	D2	D3	D4		D5	D6	D7	
Cheng 2023	*	*	*	*	**	*	*	*	Good
Ezaki 2012		*	*	*	**		*	*	Good

D1: Is the case definition adequate/Representative of the exposed cohort?

D2: Representative of the cases/Selection of the non-exposed cohort.

D3: Selection of Controls/Ascertainment of exposure.

D4: Definition of Controls/ Demonstration that outcome of interest was not present at start of study.

D5: Ascertainment of exposure/ Assessment of outcome.

D6: Same method of ascertainment for cases and controls/ Was follow-up long enough for outcomes to occur.

D7: Non-Response rate/ Adequacy of follow up of cohorts.

Supplementary Table 3: GRADE assessment for main outcomes.

Certainty assessment							№ of patients		Effect		Certainty	Importance
№ of studies	Study design	Risk of bias	Inconsistency	Indirectness	Imprecision	Other considerations	Probiotic + Antibiotics	Antibiotics alone	Relative (95% CI)	Absolute (95% CI)		
**Postoperative infections**												
5	randomized trials	not serious	not serious	not serious	not serious	none	12/90 (13.3%)	25/93 (26.9%)	**RR 0.56** (0.28 to 1.10)	**118 fewer per 1,000** (from 194 fewer to 27 more)	⊕⊕⊕⊕ High	CRITICAL
**Bifidobacterium**												
2	non-randomized studies	not serious	serious[a]	not serious	not serious	none	80	76	-	SMD **0.84 SD higher** (0.51 higher to 1.17 higher)	⊕◯◯◯ Very low[a]	IMPORTANT
**Lactobacillus**												
2	non-randomized studies	not serious	serious[a]	not serious	not serious	none	80	76	-	SMD **0.48 SD higher** (1.27 lower to 2.24 higher)	⊕◯◯◯ Very low[a]	IMPORTANT
**Hospital length of stay (assessed with: Days)**												
3	randomized trials	not serious	not serious	not serious	not serious	none	95	83	-	MD **0.2 lower** (1.31 lower to 0.91 higher)	⊕⊕⊕⊕ High	IMPORTANT
**CRP (positive) (assessed with: mg/dL)**												
2	non-randomized studies	not serious	serious[b]	not serious	not serious	none	13/48 (27.1%)	29/42 (69.0%)	**RR 0.42** (0.26 to 0.68)	**400 fewer per 1,000** (from 511 fewer to 221 fewer)	⊕◯◯◯ Very low[b]	IMPORTANT

CI: confidence interval; **MD:** mean difference; **RR:** risk ratio; **SMD:** standardised mean difference

a. There is a discrepancy in the units of measurement between the two studies. Orłowska 2021 reports counts in CFU/mL, whereas Cheng 2023 uses log CFU/g.

b. Substantial heterogeneity ($I^2 > 50\%$, $p < 0.01$).

Supplementary Figure 1: Funnel plot for postoperative infection outcome.

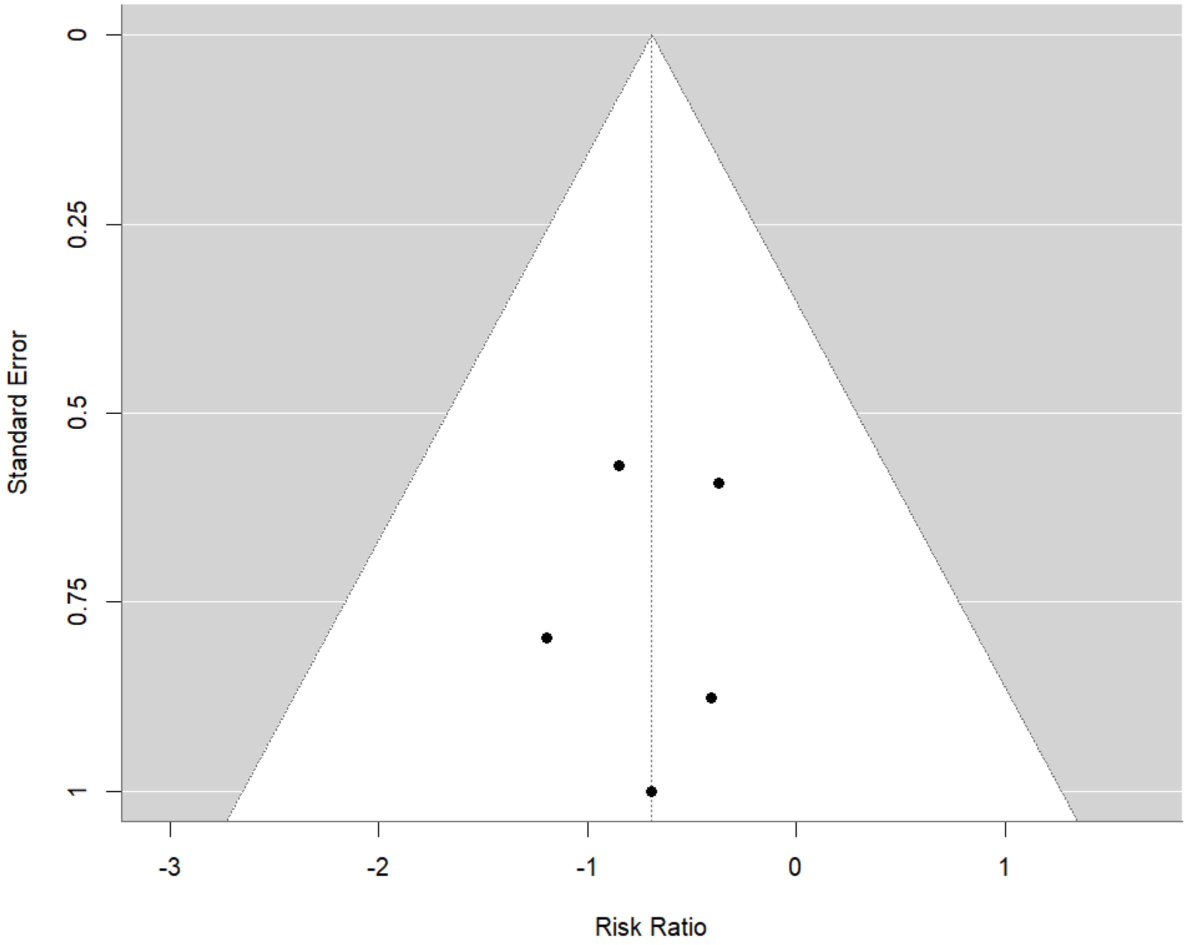

Supplementary Figure 2: Subgroup analysis for postoperative infection outcome according to the timing of probiotic initiation

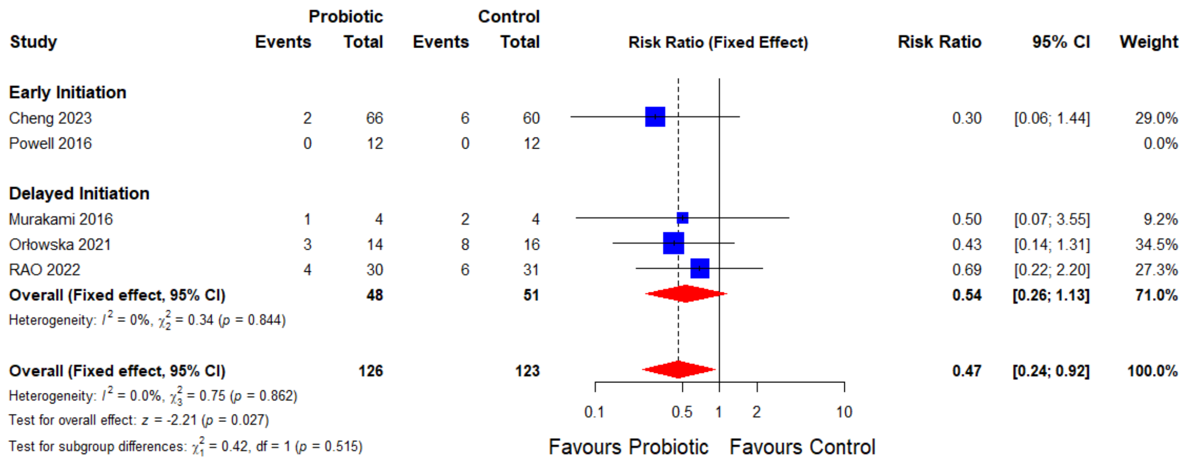

Supplementary Figure 3: Subgroup analysis for postoperative infection outcome according to the age group.

Study	Probiotic Events	Probiotic Total	Control Events	Control Total	Risk Ratio	95% CI	Weight
**Older children**							
Cheng 2023	2	66	6	60	0.30	[0.06; 1.44]	25.5%
Enayet 2022	2	30	3	30	0.67	[0.12; 3.71]	12.2%
Orłowska 2021	3	14	8	16	0.43	[0.14; 1.31]	30.3%
**Overall (Fixed effect, 95% CI)**		**110**		**106**	**0.42**	**[0.19; 0.95]**	**67.9%**
Heterogeneity: I^2 = 0%, χ^2_2 = 0.44 (p = 0.801)							
**Neonates**							
Murakami 2016	1	4	2	4	0.50	[0.07; 3.55]	8.1%
Powell 2016	0	12	0	12			0.0%
RAO 2022	4	30	6	31	0.69	[0.22; 2.20]	23.9%
**Overall (Fixed effect, 95% CI)**		**46**		**47**	**0.64**	**[0.24; 1.74]**	**32.1%**
Heterogeneity: I^2 = 0%, χ^2_1 = 0.08 (p = 0.783)							
**Overall (Fixed effect, 95% CI)**		**156**		**153**	**0.49**	**[0.26; 0.92]**	**100.0%**
Heterogeneity: I^2 = 0.0%, χ^2_4 = 0.87 (p = 0.929)							
Test for overall effect: z = -2.22 (p = 0.027)							
Test for subgroup differences: χ^2_1 = 0.40, df = 1 (p = 0.528)							

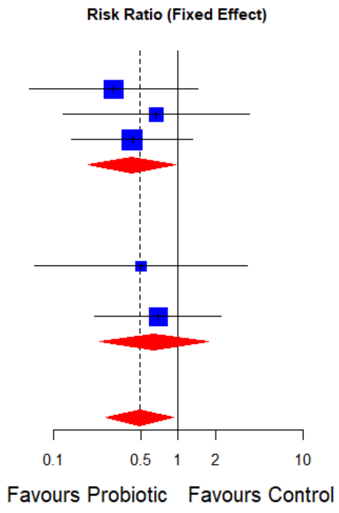

Supplementary Figure 4: Sensitivity analysis for postoperative infection outcome according to study design.

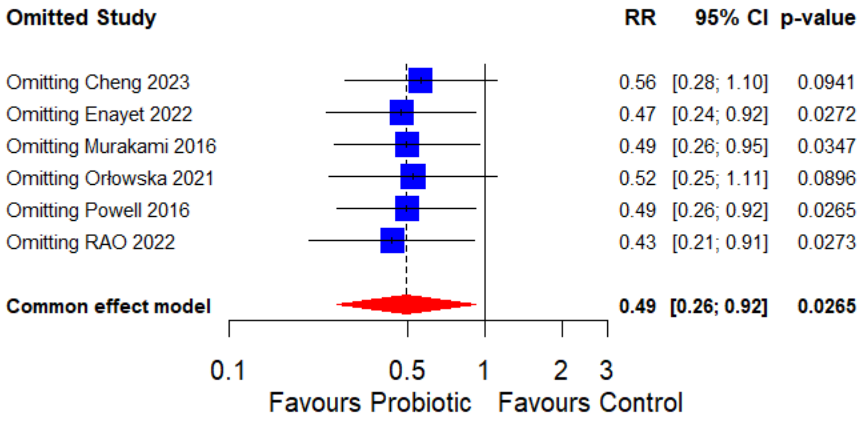

Supplement: Supplementary file 1 [file life-16-00569-s001.zip › life-4131791-supplementary.pdf]
